# Genetic Engineering of *Trypanosoma (Dutonella) vivax* and *In Vitro* Differentiation under Axenic Conditions

**DOI:** 10.1371/journal.pntd.0001461

**Published:** 2011-12-27

**Authors:** Simon D'Archivio, Mathieu Medina, Alain Cosson, Nathalie Chamond, Brice Rotureau, Paola Minoprio, Sophie Goyard

**Affiliations:** 1 Laboratoire des Processus Infectieux à Trypanosoma, Department of Infection and Epidemiology, Paris, France; 2 Laboratoire de Cristallographie et RMN Biologiques - Université Paris Descartes France, CNRS UMR 8015, Paris, France; 3 Unité de Biologie Cellulaire des Trypanosomes, CNRS URA 2581, Department of Parasitology, Paris, France; Institute of Tropical Medicine, Belgium

## Abstract

*Trypanosoma vivax* is one of the most common parasites responsible for animal trypanosomosis, and although this disease is widespread in Africa and Latin America, very few studies have been conducted on the parasite's biology. This is in part due to the fact that no reproducible experimental methods had been developed to maintain the different evolutive forms of this trypanosome under laboratory conditions. Appropriate protocols were developed in the 1990s for the axenic maintenance of three major animal *Trypanosoma* species: *T. b. brucei*, *T. congolense* and *T. vivax*. These pioneer studies rapidly led to the successful genetic manipulation of *T. b. brucei* and *T. congolense*. Advances were made in the understanding of these parasites' biology and virulence, and new drug targets were identified. By contrast, challenging *in vitro* conditions have been developed for *T. vivax* in the past, and this *per se* has contributed to defer both its genetic manipulation and subsequent gene function studies. Here we report on the optimization of non-infective *T. vivax* epimastigote axenic cultures and on the process of parasite *in vitro* differentiation into metacyclic infective forms. We have also constructed the first *T. vivax* specific expression vector that drives constitutive expression of the luciferase reporter gene. This vector was then used to establish and optimize epimastigote transfection. We then developed highly reproducible conditions that can be used to obtain and select stably transfected mutants that continue metacyclogenesis and are infectious in immunocompetent rodents.

## Author Summary

*Trypanosoma vivax* is a major parasite of domestic animals in Africa and Americas. Most studies on this parasite have focused on gathering epidemiological data in the field. Studies on its biology, metabolism and interaction with the host immune system have been hindered by a lack of suitable tools for its maintenance *in vitro* and its genetic engineering. The work presented herein focused on determining axenic conditions for culturing and growing insect (epimastigote) forms of *T. vivax* and prompting their differentiation into metacyclic forms that are infectious for the mammalian host. In addition, we describe the development of appropriate vectors for parasite transgenesis and selection *in vitro* and their use in analyzing genetically modified parasite lines. Finally, we report on the construction of the first *T. vivax* recombinant strain that stably expresses a foreign gene that maintains its infectivity in immunocompetent mice. Our work is a significant breakthrough in the field as it should lead, in the future, to the identification of parasite genes that are relevant to its biology and fate, and to work that may shed light on the intricacies of *T. vivax–*host interactions.

## Introduction

*Trypanosoma vivax* and *Trypanosoma congolense* are the main parasite species responsible for Animal African Trypanosomosis (AAT) or *Nagana*. This disease causes about 3 million deaths annually and has a marked impact on agriculture in sub-Saharan and South American endemic countries, leading to annual livestock production losses of about 1.2 billion US dollars [1]–[3]. *T. vivax* accounts for up to half of total AAT prevalence in West Africa where it is considered a predominant pathogen for domestic animals [2], [3]. The main symptoms in cattle correspond to weight loss, high abortion rates, decreased milk production, and reduced draught power and endurance [2], [3]. *T. vivax* presents a short and simple life cycle in contrast to *T. brucei* [4] and to a lesser extend to *T. congolense*. In tsetse flies, *T. vivax* development takes place in the proboscis where bloodstream forms (BSF) evolve to epimastigotes, a non infective, replicative form. After a multiplication phase, these epimastigotes undergo metacyclogenesis and transform into metacyclic infective forms, and here it is noteworthy that *Glossina spp.* are the only vectors in which *T. vivax* is able to multiply and pursue its differentiation into metacyclic forms. West African *T. vivax* populations have been introduced into South American countries - devoid of the tsetse fly - where they are now a real threat since they can be efficiently transmitted across vertebrate hosts by other hematophagous insects, including tabanids. In this case the parasites are transmitted mechanically between vertebrate hosts in a noncyclical manner, i.e. with no growth or multiplication in the insects [5], [6]. This simpler lifecycle enables *T. vivax* to adapt to different vectors and hosts and may explain why it has emerged so rapidly in South America.

Despite the fact that *T. vivax* has a major impact on emerging economies, limited efforts have gone into its study during the last decade. For our part, we have recently developed *in vivo* laboratory models of *T. vivax* infection, we initiated a detailed assessement of its infectious processes and characterized some of the key players in the immunopathology of experimental trypanosomosis [7], [8]. Our work showed that sustained and reproducible infections can easily be obtained using C57BL/6, BALB/c and Outbred mice that reproduce the parasitological, histological and pathological parameters of the livestock infection found in the field. These experimental *in vivo* models are useful in work conducted to explore the immunobiology of *T. vivax* infection and are essential in efforts made to elucidate, for instance, the function of some virulence factors *in vivo* [9], [10].

Over the last decade, recombinant gene technology has expanded our ability to investigate gene expression and function in trypanosomatids. However, transgenesis and the selection of recombinant mutants depend on our ability to maintain and grow trypanosomes in axenic cultures. The growth of insect forms of *T. vivax in vitro* was firstly described by Trager in 1959 and in the mid 1970s, in the presence *of tsetse* tissues [10], but the cultures were not stable and parasites did not survive for more than 18 days. Later, Isoun and Isoun took *T. vivax* BSF from infected cattle and managed to transform these into epimastigote forms without using insect or mammalian tissues. Unfortunately, dividing parasites were unable to withstand subculturing [11]. New methods initially dependent on feeder layer cells and subsequently adapted for the axenic cultivation of epimastigote and metacyclic forms of *T. vivax* were later proposed by several groups in the eighties and the nineties [12]–[18]. But presently, a general consensus among *T. vivax* researchers involves the difficulties to maintain the parasite in culture using the principles described in these pioneer reports [19]. This raises some concerns about the composition of the culture media described. Furthermore, this lack of a robust and efficient method for maintaining the parasite *in vitro* may readily explain the total absence of any genetic tools for engineering *T. vivax,* and this in turn has made it difficult to analyze parasite gene expression and function.

We describe herein the successful development and standardization of *in vitro* axenic cultures of epimastigote forms of *T. vivax* obtained from BSF of the IL 1392 parasite strain stably kept *in vivo* [8]. This West African stock of *T. vivax* is derived from the Nigerian isolate Zaria Y486 which is infective for rodents and can be cyclically and/or mechanically transmitted [20], [21]. Cultured epimastigote forms continue their differentiation *in vitro* into metacyclic parasites and thus acquire infectious properties in mice. In addition, we describe the first integrative expression vector for *T. vivax*, designed to constitutively express foreign gene products and bearing the neomycin phosphotransferase (NeoR) selectable marker which confers resistance to G418. This expression system also harbors a long ribosomal promoter region of *T. vivax* to drive transcription of the reporter and NeoR genes and thus improve gene expression and permit recombinant selection.

We used this vector to establish conditions conducive to the efficient and highly reproducible transfection and selection of *T. vivax* epimastigote mutants. We show here that the p*Tv*LrDNA-Luc plasmid is appropriately integrated and that the product of the reporter gene is expressed at detectable levels. Finally, the culture protocols described herein were used successfully for the *in vitro* selection, growth and development of all the evolutive forms of genetically engineered *T. vivax* that are infectious to immunocompetent mice.

## Materials and Methods

### Ethics statement

All mice were housed in our animal care facilities in compliance with European animal welfare regulations. Institut Pasteur is a member of Committee #1 of the *Comité Régional d'Ethique pour l'Expérimentation Animale* (CREEA), Ile de France. Animal housing conditions and the protocols used in the work described herein were approved by the *“Direction des Transports et de la Protection du Public, Sous-Direction de la Protection Sanitaire et de l'Environnement, Police Sanitaire des Animaux”* under number B 75-15-28, in accordance with the Ethics Charter of animal experimentation that includes appropriate procedures to minimize pain and animal suffering. PM is authorized to perform experiments on vertebrate animals (license #75–846 issued by the Paris Department of Veterinary Services, DDSV) and is responsible for all the experiments conducted personally or under her supervision as governed by the laws and regulations relating to the protection of animals.

### *T. vivax* parasite strain and *in vivo* maintenance

*Trypanosoma (Dutonella) vivax* IL 1392 was originally derived from the Zaria Y486 Nigerian isolate [8], [22]. These parasites had recently been characterized and were maintained in the laboratory by continuous passage in mice, as previously described [8]. Seven to 10-week-old male Swiss Outbred (CD-1, RJOrl:SWISS) or BALB/c mice (Janvier, France) were used in all experiments. Mice were injected intraperitoneally with bloodstream forms of *T. vivax* (10^3^ parasites/mice) or with cells derived from axenic cultures (2×10^6^ metacyclic-like trypomastigotes). Parasitemia was determined as previously described [9]. All animal work was conducted in accordance with relevant national and international guidelines (see above).

### Parasite maintenance in axenic cultures and *in vitro* differentiation

Epimastigote cultures were initiated with the blood of infected mice once parasitemia reached at least 5.10^8^ parasites/ml. Blood was collected by cardiac puncture onto heparin (2500 IU/kg), and was then diluted 1 ∶ 8 (v/v) with PBS −0.5% glucose to 5.10^7^ parasites/ml. Parasites were separated from red blood cells by differential centrifugation using a swingout rotor (Jouan GR412, Fisher Bioblock Scientific, Strasbourg, France). This technique offered a higher index of recovery of viable BSF (4.0–4.5×10^8^ BSF, corresponding to 80–90% recovery) than classic ion-exchanged chromatography using DEAE-cellulose based methods. Briefly, diluted blood was processed by one first round of centrifugation (5 minutes at 200 g) and the supernatant withdrawn with a Pasteur pipette without disturbing the red blood cell layer and the thin interface containing the white blood cells. Parasite-enriched suspension was submitted to a second round of centrifugation (10 minutes at 300 g). Supernatant was then centrifuged 10 minutes at 1800 g and BSF - containing pellets devoid of host cells used to inoculate culture flasks containing different culture medium to a final concentration of 10^6^ to 10^7^ parasites per ml. These were then incubated at 27°C in an atmosphere devoid of CO_2_ (see Table 1 for details). Parasite adhesion was checked by visual inspection after 4 to 5 days when half the media had to be changed. Cultures were maintained in 25 cm^2^ polystyrene flasks (T25) (Corning, Bagneaux-sur-Loin) by changing 3 ml of medium every 2 or 3 days. The TV_1–5_ media used in this study were based on D-MEM (Dulbecco's Modified Eagle's Medium, Invitrogen) or IMDM (Iscove's Modified Dulbecco's Medium, Invitrogen). These media were supplemented with 0–0.4% glucose, 0–20% heat-inactivated fetal calf serum (FBS, MP Biomedicals or Invitrogen) and/or 0–20% heat-inactivated goat serum (GS, Invitrogen), 0.03 mM bathocuproinedisulfonic acid, 0.45 mM L-cysteine, 0.2 mM hypoxanthine, 0.14 mM ß-mercaptoethanol, 0.4–6 mM L-proline, 0.05 mM thymidine, and 25 mM HEPES pH7.4, as indicated in Table 1. All supplements were obtained from Sigma Aldrich except HEPES (Invitrogen, Cergy Pontoise).

**Table 1:** Parasite culture media.

Medium	HMI107	B	TV1	TV2	TV3	TV4	TV5
Base	IMDM	IMDM	IMDM or DMEM	IMDM or DMEM	IMDM and DMEM	DMEM	IMDM
**FCS (%)**	20	20	20	-	10	10	10
**GS (%)**	-	-	-	20	10	10	10
**Glucose (%)**	0.4	0.4	0.4	-	0.2	0.2	0.4
**L-Proline (mM)**	0.6	6	2	2	1–4	2	2

Conditioned medium consisted of 1 volume of centrifuged (10 minutes at 1800 g) and filtered supernatant from 2- to 3-week-old cultures, diluted with 2 volumes of fresh medium.

### Fluorescence microscopy

Parasites from the supernatant or from the adherent layer were collected, washed in PBS and resuspended at 5.10^7^/mL. 40 µL of the various suspensions were spotted onto coverlips and allowed to settle for 10 min before being fixed for 15 s in cold methanol. Slides were incubated for 1 h at 37°C with mouse monoclonal antibodies directed against paraflagellar rod protein 2 (anti-PFR2 L8C4) [23]. They were then washed 5 times with PBS and incubated for 45 min with a goat anti-mouse IgG secondary antibody labeled with Alexa Fluor 488 (MolecularProbes, France). DNA was stained with 3 µg/mL 4′,6′-diaminido-2-phenylindole (DAPI, Sigma-Aldrich) for 10 min at room temperature and the slides were washed 5 times and finally mounted in Fluoromount G (Interchim, Montluçon). Parasite forms were examined under an Olympus immunofluorescence multifilters BH-2 UV (Zeiss) or DMR (Leica) microscope. Images were captured, for instance using a CoolSnap HQ camera (Roper Scientifique).

### Construction of the p*Tv*LrDNA-luc vector

Several steps were required to construct the first *T. vivax* specific vector (see Table 2 for primer sequences). Initially, *Tv*PRAC 5′UTR sequence containing the Spliced leader Acceptor Site (p.-582 to p.-1) was amplified from BSF *T. vivax* genomic DNA using SLasF 5′ and SLasRmcs multiple cloning site primers. The amplified product (617 bp) was subcloned into pCR Blunt topo vector (Invitrogen); this construct was submitted to nested PCR using SLasKpnI-F and McsSacI-R primers to introduce specific *KpnI* and *SacI* sites. A 616 bp fragment was then obtained after *KpnI* and *SacI* digestions and appropriately inserted into pBlueScript KS to create p*Tv*5′UTRa. The *T. vivax* intergenic region between α and β tubulin (*Tv*tubαβ) was amplified from BSF genomic DNA using *Tv*TubαβBam-F and *Tv*TubαβAsc-R primers. The fragment obtained (506 bp) was digested with *BamHI* and *AscI* and inserted into *BamHI* and *AscI* sites of the p*Tv*5′UTRa vector. The neomycin resistance gene cassette (NeoR) was excised from pXS2-GFP [24], by digestion with *AscI*/*PacI* and the 802 bp Neo-fragment further inserted downstream of *Tv*Tubαβ to produce vector p*Tv*5′UTRb. In order to provide a putative 3′ polyadenylation signal for the *NeoR* gene, the 330 bp intergenic region located between *Tv*Tub β and *Tv*Tub α was amplified by PCR using *Tv*TubβαF and *Tv*TubβαSac-R and the resulting fragment was digested with *PacI*/*SacI* and subcloned into the *PacI*/*SacI* sites of the p*Tv*5′UTRb digested vector. Firefly luciferase reporter gene was purified from *Trypanosoma brucei* pLEW100 vector [25], digested with *HindIII* and *BamHI* and cloned among the *Tv*PRAC 5′UTR and *Tv*Tubαβ sequences of p*Tv*5′UTRb to produce p*Tv*-LUC. Finally, 2 derivatives of p*Tv-*LUC were constructed containing a long (1.8 kb, LrDNA) or a short (1.2 kb, SrDNA) upstream of the 18S rDNA sequence. These putative RNA PolI promotor regions were amplified with *Tv*rDNAK-F and *Tv*rDNAK-R primers and further inserted into the *KpnI* site of p*Tv-*Luc to produce the final expression vectors p*Tv*LrDNA-Luc and p*Tv*SrDNA-Luc. All steps in these constructions were validated by sequencing to check that the different fragments were in the correct location and orientation.

**Table 2:** Primer sequences.

SLasF	5′- GAGCTCGGTAGGGAGGCGATACC- 3′
SLasRmcs	5′- GGTACCTTAATTAAGGCGCGCCGGATCCTCTA GAGAATTCAAGCTTCTCAACAACGCGC- 3′
SLasKpnl-F	5′- GCGGTACCGGTAGGGAGGCGATACC- 3′
McsSacI-R	5′- GCGAGCTCTTAATTAAGGCGCGCCGGATCC- 3′
*Tv*tubαβBam-F	5′- GCCGGATCCACGCCCCGTTGTTGCGGGCC- 3′
*Tvt*ubαβAsc-R	5′- CGGGCGCGCCATTCGCTTGGGTTTTCTTGG- 3′
*Tv*tubβα-F	5′- CGTTAATTAAACGACGCCCACTTCCCCACC- 3′
*Tv*tubβαSac-R	5′- CGGAGCTCGACGGACCGAAGGAGTTCG- 3′
*Tv*rDNAK-F	5′- GCGGTACCGAGGAGCTGATTTCGCCACTGC- 3′
*Tv*rDNAK-R	5′- GCGGTACCGCTTCACTTGATGATCGTTTCG- 3′
upFrProm	5′- CGCGTGCTTGCCGAGCGCCGCGTGT- 3′
upRrProm	5′- GTCTCTGTTCAAAATTAATGGTATCGCCTC- 3′
downFrProm	5′- CGGCCAGTGAATTGTAATACGACTC- 3′
downRrProm	5′- GCGAGTGAGGGGGCGGCAGGCGCA- 3′

### p*TvLrDNA-*GFP vector construction

The GFP cassette was excized from the vector *pXS2-GFP* [24] by *HindIII* and *EcoRI* digestion. The resulting gene fragment (714 bp) was used to replace the firefly luciferase reporter gene of the *pTvLrDNA-Luc* vector between the *TvPRAC* 5′UTR sequence (*HindIII* site) and the intergenic region *Tvtubαβ* (*EcoRI* site). As here above, appropriate replacement was validated by sequencing.

### Trypanosome transfection

Parasites were recovered from the flasks after 15 to 20 days of culture. In order to recover adherent parasites without causing physical damage, the flasks were washed twice with PBS −0.5% glucose and adherent cells were detached from the surface of the plastic using a cell scraper in the presence of PBS.

For transfection using the Gene Pulser system (Biorad, Marnes-la-Coquette), parasites were washed and resuspended in Cytomix at 0.5–1.5×10^8^ cells/ml then 500 µl suspensions were mixed with 5–20 µg vector DNA and electroporated in a 4 mm gap cuvette using two consecutive pulses of 1.2–1.8 kV, 200 Ω resistance and 50 µF capacitance. For the Amaxa nucleofections, pellets containing 0.5–1.5×10^8^ parasites were resuspended in 100 µl of Human T Cell solution (Lonza, Levallois Perret), mixed with 20 µg of circular or linearized plasmids and subjected to nucleofection using the 5 different Amaxa programs. The total volume of transfections was adjusted to 3 ml with TV3 medium and incubated at 27°C. Forty eight hours after transfections, G418 (Invitrogen, Life Technologies, Villebon sur Yvette) was added to the cultures at a final concentration of 0.5 µg/ml to allow selection of recombinant *T. vivax*. Genetically engineered parasites, whose resistance to G418 was confered by the Neo gene, were selected over time when a massive cell death was observed in the cultures leaving colonies of stably transformed *T. vivax* behind (generally after 10 days). Total parasite genomic DNA was prepared from *in vitro* cultures with pure link genomic DNA (Invitrogen, Life Technologies, Villebon sur Yvette). Correct plasmid integrations were checked by PCR using standard techniques and upFrProm/upRrProm or downFrProm/downRrProm oligonucleotides pairs and Dream Taq polymerase (Fermentas, Villebon sur Yvette, France).

### *In vitro* luciferase assay

A luciferase assay kit (Roche Molecular Biochemicals; Mannhein, Germany) was used to monitor luciferase expression. Serial dilutions of parasite suspensions were washed in PBS and pellets were resuspended in 150 µl of cell lysis buffer. Debris was removed by centrifugation. The lysates were then transferred into white, 96-well microplates (Dynex Technologies, Chantilly, France). Light emission was initiated by adding the luciferin-containing reagent, in accordance with manufacturer instructions. The plates were immediately transferred to the luminometer (Berthold XS^3^ LB960) and light emission measured for 0.1 s. Luminescence was expressed as Relative Light Units (RLU).

### Flow cytometry

Wild type or *Tv*GFP parasites were recovered from 14 days axenic cultures in TV3 medium. Adherent and supernatant cell populations were washed and resuspended in PBS −0.5% glucose balanced salt solution (2×10^6^ cells/ml) containing 1 µg/ml of propidium iodide. Two-color acquisition was carried out with a FACScalibur cytofluorometer (Becton Dickinson). Dead cells were excluded from the analysis by gating out propidium iodide-stained cells. Parasites were gated on forward-light scatter/side-light scatter combined gate, and 40000 events were acquired. Results were analyzed by FlowJo software (Tree Star, Inc).

## Results

### Establishment of *T. vivax* axenic cultures and impact of serum source

**Figure 1:**
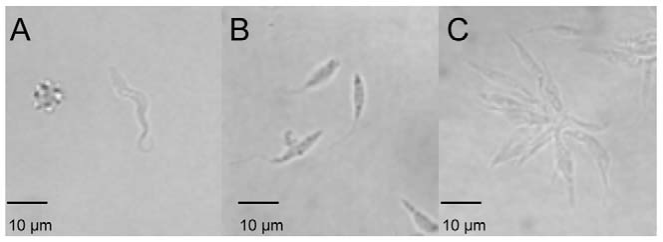
Establishment of axenic cultures of *T. vivax* epimastigotes. BSF (A), BSF-derived epimastigotes (B), Epimastigotes forming rosettes in the culture supernatants (C).

We started *T. vivax* adaptation to axenic cultures using parasites previously adapted *in vivo* in mice [8]. More specifically, we began by inoculating HMI107 or B media with BSF purified from infected mice and incubating the preparations at 27°C, as described by Hirumi et al. in 1991 or by Gumm, in 1991, respectively [14], [15]. Various protocols were tested, e.g. different BSF levels, different concentrations of the various amino acids, different pH values, temperatures, reducing agents, and type of flask, but none of the cultures developed. Since the absence of glucose had previously been described as a factor triggering *T. brucei* BSF differentiation into procyclic forms [26], we used purified bloodstream forms of *T. vivax* to inoculate TV1 and TV2 media that varied in composition mainly in terms of serum nature and/or the presence of glucose (see Table 1 for details). BSF parasites were observed to be highly mobile for the first 3 days of cultivation in both TV1 and TV2 media. By day 4, some parasites started to attach to the surface of the plastic and showed some of the morphological changes commonly seen in BSF that are differentiating into epimastigote forms, as previously described by Gumm [14]. Differentiating parasites replaced the prominent undulating membrane by a flagellum that emerges from the anterior portion of a shorter body and an anterior kinetoplast. However, the parasites were still unable to divide, and this in both TV1 and TV2 media, and they died after 7 days or 14 days, respectively. When grown in TV3 medium - that contains an equivalent mixture of complete IMDM and DMEM media (vol/vol) - BSF attached to the plastic flask after 4–5 days, suggesting that they were engaged in the process of differentiation into epimastigote cells. Three days later (day 7/8 of culture), some parasites were seen to have shortened, indicating that they had differentiated into epimastigotes. Even more importantly, they started to multiply by forming small clusters (Figure 1 A–C). These clusters increased in number and size and 3 to 4 weeks later had covered the entire surface of the culture flask. At this stage, both rosettes and free-swimming cells (1.5.10^7^ cells/flask) became abundant in the supernatant and these parasites could then be used to inoculate new flasks.

In order to determine whether the mixture of the two sera and/or medium composition was critical for growth, parasites obtained from the TV3 culture supernatant were used to inoculate fresh flasks containing IMDM or DMEM supplemented with 10% FCS, 10% GS and different concentrations of glucose (TV4 and TV5 media, respectively), comparable to those in TV1 and TV2 media. Only IMDM-based medium (TV5) supported *T. vivax* growth at similar kinetics to TV3 medium, and this regardless of the glucose concentration. Different combinations (vol/vol) of five batches of fetal calf sera and three batches of goat sera were able to support growth without any significant differences (data not shown), indicating that the positive effect of goat serum on *T. vivax* growth is not an artifact due to serum batch heterogeneity. Conversely, parasites were unable to grow in media supplemented solely with 20% FCS or 20% GS. Medium TV3 was therefore chosen for all subsequent cultures and experiments.

### Characterization of culture stages and epimastigote *in vitro* differentiation

**Figure 2:**
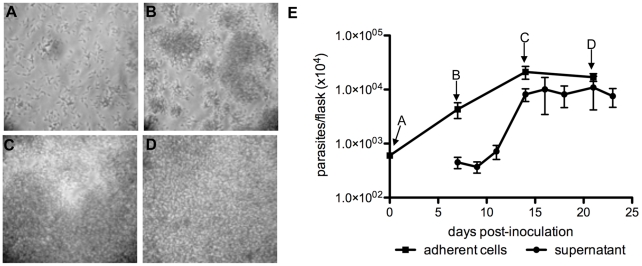
Kinetics of *T. vivax* growth *in vitro*. Microscopic examination of adherent cells 2 hours (A), 7 days (B), 14 days (C) and 21 days (D) after inoculation with 1.5.10^7^ parasites. 100× magnification. Parasite numbers in ongoing cultures (E). Results are expressed as arithmetic means ± SD.

Parasite growth kinetics and metacyclogenesis were investigated to characterize the parasite stages observed during axenic culture. As can be seen in Figure 2, TV3 medium was inoculated with 1.5.10^7^ cultured parasites and their development was monitored for three weeks. The parasites attached to the surface of the plastic within 2 hours (Figure 2A) and formed micro-colonies after 7 days (Figure 2B). Conspicuous parasite multiplication was then observed for the following week and cells completely covered the entire surface of the plastic between days 14 and 21 (Figure 2C and 2D). Parasite cell numbers were determined in the supernatant and the number of adherent cells was evaluated after scraping. As shown in Figure 2E, the number of cells in the supernatant increased with time and in proportion to the density of the adherent cell layer. A confluent 25 cm^2^ flask was able to produce 5.10^7^ to 1.10^8^ parasites in the supernatant every two days. With appropriate care and a medium amendment (see below), it was possible to conserve parasite viability in a single flask for 6 to 10 weeks. In addition, the supernatants provided a sufficient number of parasites to initiate new cultures and thus support regular *in vitro* passages weekly or every 2–3 weeks.

**Figure 3:**
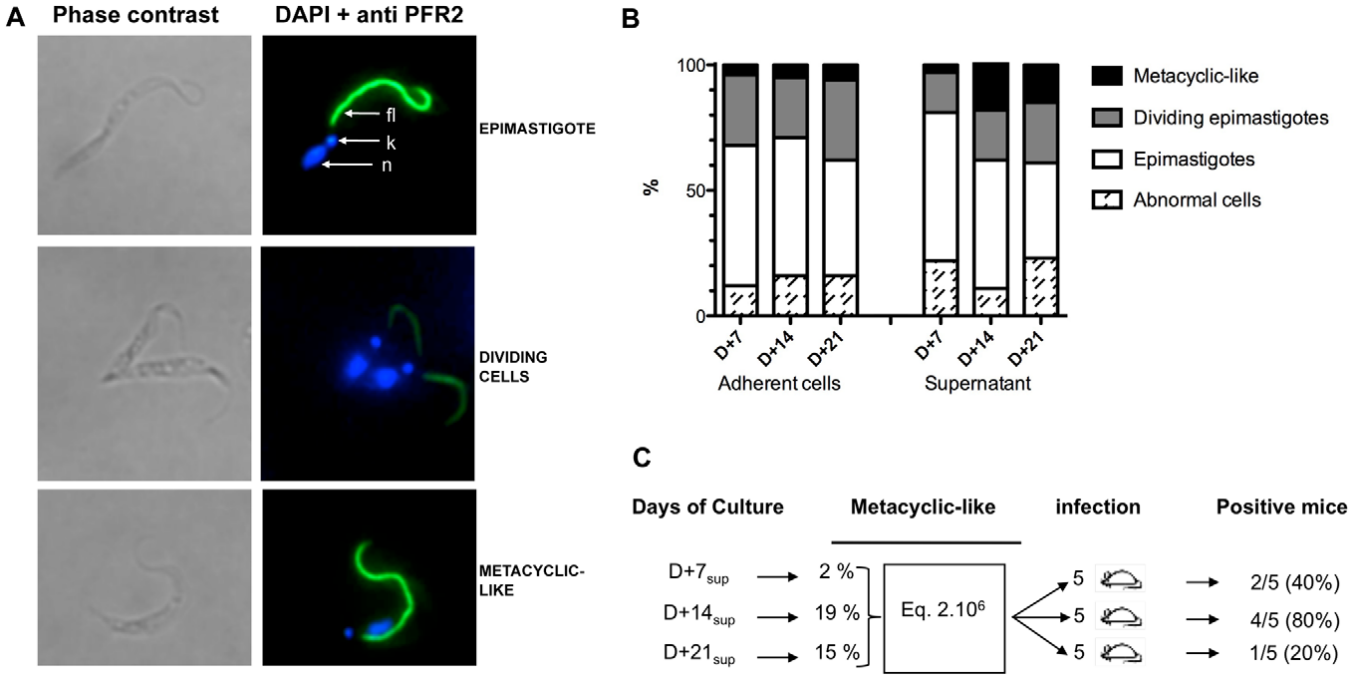
Characterization of *T. vivax* developmental stages *in vitro*. Supernatant and adherent layer parasites taken from ongoing axenic cultures were labeled with anti-PFR2 antibodies (see Methods), stained with DAPI and examined under an epifluorescence microscope: n = nucleus; k = kinetoplast; fl = flagellum (A). Bars represent relative numbers in each parasite population (B). Metacyclic-like parasites in culture supernatants were quantified by IMF on days 7, 14 and 21. An equivalent number of metacyclic-like trypomastigotes (2×10^6^) were taken at each time point and injected intraperitoneally in groups of 5 Balb/c male mice. Parasitemia was measured individually for 28 days and the number of positive mice (and % within the group) was noted.

In efforts to determine whether established culture conditions were suitable for the differentiation of epimastigotes into infective metacyclics, we monitored the proportions of the different parasite forms in ongoing cultures. The relative positions of nuclei and kinetoplasts were evaluated by immunofluorescence after DNA staining with DAPI, and flagellum length and position were determined using antibodies to label paraflagellar rod protein 2 (PFR2) [23]. As shown in Figure 3A, the kinetoplast in epimastigote forms was located between the nucleus and the anterior part of the cell body, whereas metacyclic-like trypomastigotes had a longer body-attached flagellum and a kinetoplast posterior to the nucleus. Numerous epimastigotes in the cultures were also observed to be dividing. Changes in parasite forms present in the supernatant and in the adherent layer were monitored throughout the culture period and the populations in each developmental stage were quantified on culture days 7, 14 and 21. We observed that the different populations that made up the adherent layer did not change in proportion over time, with the vast majority (about 74%) of cells consisting of epimastigotes throughout the plastic colonization period (Figure 3B). The total proportion of epimastigotes was stable throughout the culture and only 24 to 32% were actively dividing cells. Some metacyclic-like cells were also observed in the population of attached cells, but accounted for only a small and invariant proportion (about 5%). Conversely, changes in the trypomastigote population in the supernatant suggested that an active process of epimastigote differentiation into metacyclic cells (metacyclogenesis) was taking place. For instance, the parasite population in the supernatant after 7 days was similar to that observed in the adherent layer. A substantial change then occurred from day 14 with a dramatic increase in the proportion of metacyclic-like parasites (3 to 19%). This was accompanied by a considerable decrease in the number of epimastigotes and abnormal cells in the supernatant. The population of metacyclic-like cells peaked at this point then decreased by day 21.

The virulence of these axenic trypomastigotes was assessed by first collecting parasite populations from ongoing culture supernatants and analyzing these by immunofluorescence. The results showed that 2%, 19% and 16% of metacyclic-like cells were present on days 7, 14 and 21, respectively. Equivalent numbers of metacyclic-like cells (2×10^6^) were then injected intraperitoneally in 3 groups of 5 Balb/c mice and parasitemia was measured over a period of 28 days (Figure 3C). The results showed that 40%, 80% and 20% of individual blood smears contained BSF parasites (>10^4^ parasites/ml) between days 12 and 21. Since equivalent numbers of metacyclic-like cells were injected into the mice, our results indicated that the metacyclic-like cells present in the cultures were at different stages in their maturation and that the axenic differentiation process is not synchronous.

### Factors affecting *T. vivax* epimastigote adhesion and growth in axenic cultures

Marked alkalinization of the TV3 medium was observed less than 30 minutes after the epimastigotes became attached to the plastic surface (the pH increased from 7.4 to 8.6). When the parasites were left at this high pH, they adopted a round shape and died. This phenomenon was prevented by increasing the HEPES final concentration to 100 mM and in this manner the pH was held at 7.4 during the initial growth process. We observed that adherent parasites could not be easily removed from the plastic surface and attempts to use a cell scraper were unfruitful, causing death in most of the parasites. Since the pH appears to be critical for adhesion, we analyzed whether pH variations impacted on parasite attachment. Confluent flasks were washed twice with PBS at pH 7.4, pH 6 or pH 8.5. Each of the three washing conditions led to partial cell detachment (around 10^8^ cells) but the adherent cells that remained (approximately 2×10^8^) could then be easily scraped off the plastic surface without cell damage. The removal of medium (and probably serum), rendered the attached cells less cohesive and loosened cells that presented higher levels of viability. Such a procedure was employed to provide 3.10^8^ cells in a T25 confluent flask, and these were used to reinoculate fresh flasks or to carry out further experiments.

Since L-proline is well known to be an important source of carbon for trypanosomes [27], *T. vivax* epimastigote growth was also estimated at different proline concentrations (1 mM, 2 mM, 4 mM) in TV3 medium. 10^7^ cells were used to inoculate T25 flasks and the time required to obtain a confluent layer of cells was determined. As the proline concentration in the flask increased, the time required to obtain confluence decreased, from 3–4 weeks (1 mM proline) to 14 days (4 mM proline). Since variations in glucose concentrations did not affect parasite growth, the results here indicate that proline is a key player in the growth of *T. vivax* epimastigotes. Our data showed that 4 mM L-proline was the optimal concentration in TV3 medium. Higher concentrations did not significantly improve culture conditions or parasite growth and maintenance.

The effects of epimastigote density on cultivation were studied by performing a limiting dilution assay using fresh or conditioned media [28]–[30]. Here, 10^7^ to 10^4^ parasites were used to inoculate flasks containing TV3 media. The density of adherent cells and the presence of micro-colonies on the plastic surface were scored after 3 weeks of culturing at 27°C, with regular changes of the media. Epimastigotes used to inoculate fresh TV3 media, at concentrations of less than 10^6^/per T25 flask became round, were unable to multiply and died after a week. By contrast, when using conditioned media containing 30% supernatant from former *T. vivax* cultures, flasks inoculated with only 10^5^ parasites gave rise to dense parasite clusters after 4 weeks. Micro-colonies 2 mm in diameter were also observed after 4 weeks in flasks inoculated with 10^4^ cells, and these reached confluence by 6 weeks.

### Genetic tool design and transfection of *T. vivax* epimastigotes

**Figure 4:**
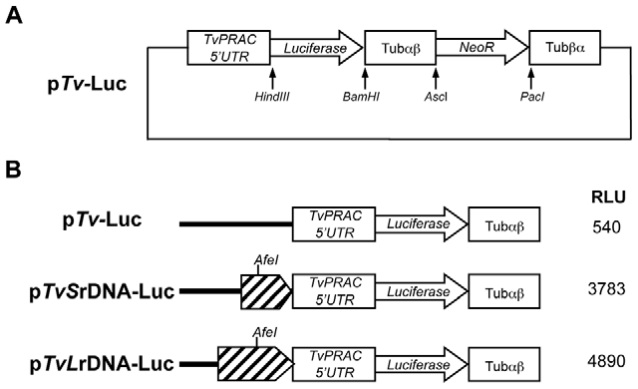
*Trypanosoma vivax*-specific luciferase vectors. Schematic representation of p*Tv*LUC vector constructs (A); *Tv*PRAC5′UTR, upstream of the *Tv*PRAC region and containing spliced leader acceptor site; Tubαβ, intergenic region between alpha and beta tubulin genes; NeoR, neomycin phosphotransferase gene; Tubβα, intergenic region between β and α tubulin genes. Representation of the ribosomal promoter region cloned into p*Tv-*Luc and associated luminescence in Relative Light Units (RLU) detected in the supernatant 48 h after epimastigote transfection with p*TvS*rDNA-Luc and p*TvL*rDNA-Luc (B).

No genetic manipulation of *T. vivax* has ever been described in the literature, nor any expression of transgenes. We therefore began by constructing plasmids containing the luciferase reporter gene (see Material and Methods) in efforts to determine appropriate conditions for reproducible transfection of *T. vivax*. Figure 4A schematically represents p*Tv-*Luc vector that was specially designed in order for *T. vivax* to express the luciferase gene and neomycin phosphotransferase (NeoR), which confers resistance to G418. Upstream of the reporter gene we cloned the 5′UTR of the *T. vivax* proline racemase gene (*PRAC*) that contains an efficient spliced donor acceptor site [9]. Work in *T. congolense and T. brucei* has previously identified RNA pol I promoter elements in regions spanning 2 kb upstream of the 18 S element of the rDNA gene cluster [31], [32]. But, taking *T. cruzi* specific vectors as an example [33], where poor gene expression is observed in the absence of such sequences, we aimed to provide the *T. vivax* vector with a hyperexpression cassette to regulate gene transcription. In order to better define the region with a putative *T. vivax* promoter, we constructed two different plasmids harboring respectively a long 1.8 kb fragment (p*TvLr*DNA-luc) and a short 1.2 kb fragment (p*TvSr*DNA-luc) upstream the 18 S rDNA gene (see Figure 4B). Axenic *T. vivax* epimastigotes were then transiently transfected with the p*Tv-*LUC, p*TvLr*DNA-luc or p*TvSr*DNA-luc plasmids using a Gene Pulser electroporator under high voltage conditions. Luciferase activity was measured in the different cell lines 48 h after transfection to check for the presence of the putative pol I promoter in selected sequences.

**Figure 5:**
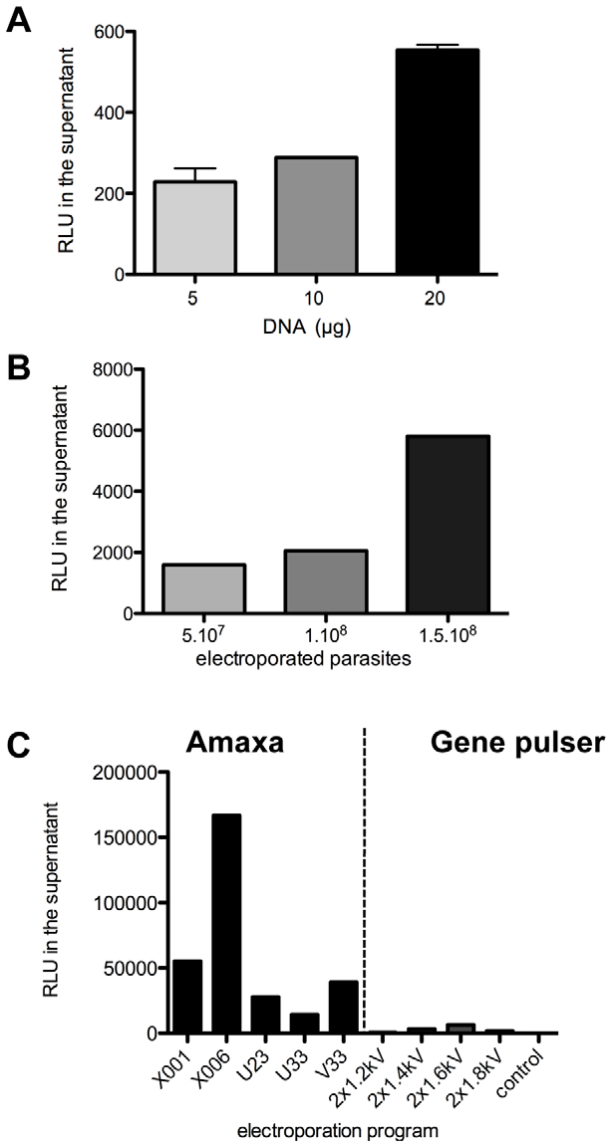
Set-up of T. vivax transfection. (A) Different doses of circular p*TvL*rDNA-Luc were used to transfect 1.10^8^ parasites using a Gene Pulser protocol (2×1.6 kV) and the quantity of light emitted by the supernatant was quantified 24 h post-transfection. Results are expressed as arithmetic means ± SD. (B) Using the same protocol, transient transfection efficiency was evaluated with different parasite loads; (C) Effect of Amaxa or Biorad Gene Pulser protocols on electroporation efficiency. Parasites taken from supernatants are checked for light emission 24 h post-transfection.

Interestingly, luciferase activity was 10 times higher after transfection with all plasmids containing the putative rDNA promoter sequence compared to transfection with the p*Tv*LUC plasmid. Comparable levels of luciferase activity were obtained with the p*TvLr*DNA-luc and p*TvSr*DNA-luc plasmids, suggesting that the rDNA promoter region in *T. vivax* is located in a 1.2 kb region directly upstream of the 18 S ribosomal DNA gene (Figure 4B). However, given that the p*TvLr*DNA-luc plasmid has a better potential for recombination, subsequent work conducted to optimize *T. vivax* transfection conditions was conducted using this vector. Different concentrations of circular plasmid molecules were tested, and 20 µg was observed to be the optimal DNA concentration for *T. vivax* transfection studies, as based on the RLU obtained and as illustrated in Figure 5A. Different numbers of axenic parasites were then electroporated with 20 µg of circular plasmid. The results obtained showed that 1.5.10^8^ parasite cells/per transfection gave the highest RLU in the supernatant (Figure 5B). Then, in order to check that an acceptable success rate was obtained with *T. vivax* transfection, the relative efficiency of the gene transfer was measured by comparing transfection using a Gene Pulser system and Amaxa nucleofection. With 20 µg of circular plasmid used in each of the transfer conditions, the epimastigotes were subjected to 4 different Gene Pulser system voltage conditions and to 5 different nucleofection Amaxa programs.

Examination of the cells after Gene Pulser electroporation showed massive rates of mortality at all voltages used (not shown). Moreover, the parasites were unable to adhere to the plastic flasks, and this precluded any growth in TV3 medium. By contrast, as described previously for *T. brucei* and *T. congolense* [34], [35], the Amaxa nucleofection method greatly enhanced transfection efficiency and additionally, *T. vivax* showed better adhesion to the plastic surface and increased survival rates. Thus, as can be seen by the expression of luciferase monitored 24 h after gene transfer, the transfection efficiency obtained with Amaxa program “×006” was 25 fold higher than that obtained with the best Gene Pulser conditions (two 1.6 kV pulses) (Figure 5C). This program was therefore used in all subsequent experiments.

### Gene integration and impact of transfection *in vitro* and *in vivo*

**Figure 6:**
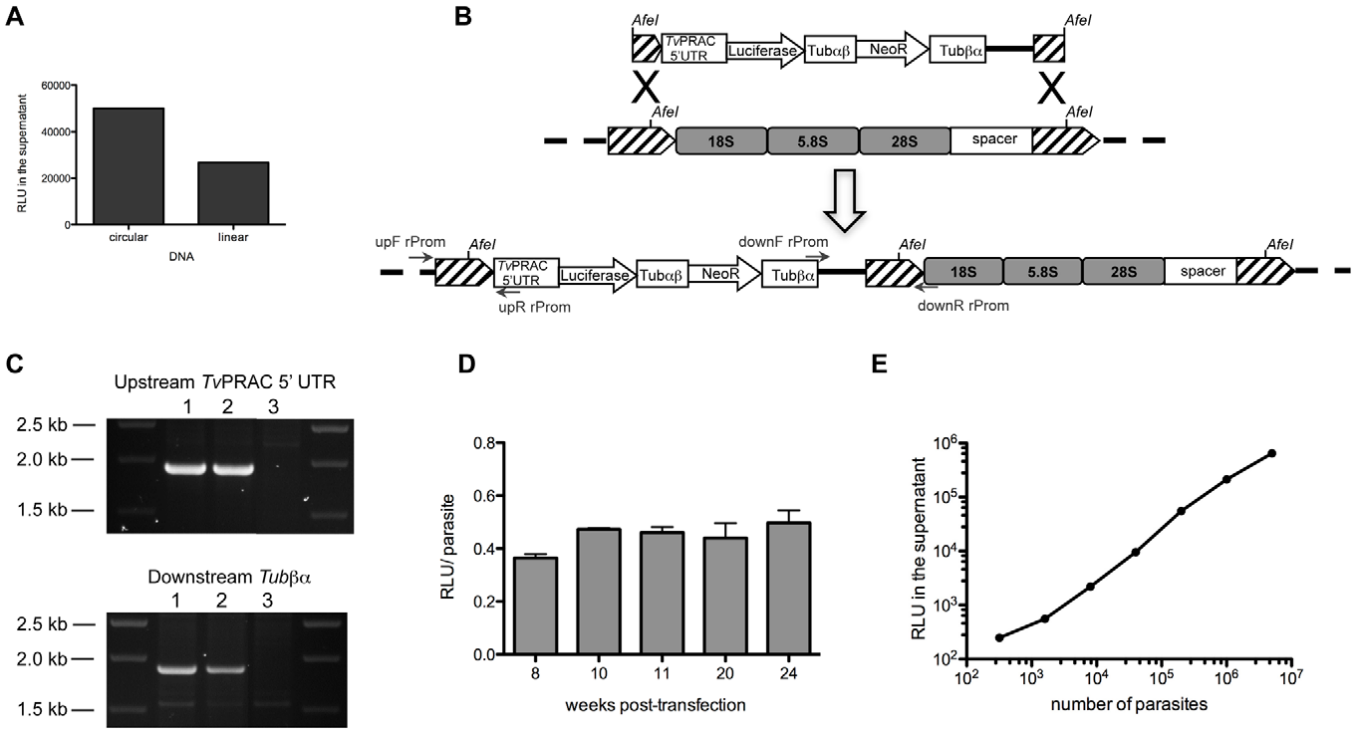
Obtaining the stably transfected *T. vivax TvLrDNA-Luc* strain. (A). *T. vivax* axenic epimastigotes were transfected with circular or *AfeI* linearized p*TvL*rDNA-Luc using the ×006 Amaxa program and luciferase light emission (RLU) was measured 24 h post-transfection in the supernatants. (B) Schematic representation of p*Tv*LrDNA-Luc linearized with *AfeI* and its expected recombination in the parasite ribosomal DNA region. Hatched boxes show ribosomal promoter-containing regions; Black crosses represent recombination events; grey arrows indicate primers used to verify plasmid integration into *T. vivax* genome. 18 S, 5.8 S and 28 S, rRNA genes. (C) Verification of the 5′- and 3′- integration of the vector. PCR using respectively upF/upR (upper panel) and downF/downR (lower panel) primers. PCR was performed on genomic DNA from 2 independent cultures stably transfected with p*TvL*rDNA-Luc (1, 2) or from WT strain (3). (D) Changes in luminescence (RLU) emitted per p*TvL*rDNA-Luc parasite after G418 selection. (E) Graphic representation of bioluminescence expressed as luciferase light emission (RLU) of increasing numbers of *TvLr*DNA-luc parasites.

Efficient selection conditions were then determined conducive to obtaining stably p*TvLr*DNA-luc transfected parasites. 10^7^ parasites were used to inoculate fresh flasks containing various concentrations of G418 (10 to 0.25 µg/ml), and TV3 medium was changed every 2 or 3 days. A G418 concentration of 0.5 µg/ml was sufficient to kill all the cells after 10 days of culture. In order to maintain the requirement for ‘quorum sensing’ on transfectant growth, cells were maintained in TV3 conditioned media in the presence of G418 for at least 4 weeks. In order to obtain stably transfected parasites, we targeted the *T. vivax* ribosomal region by using *Afe*I linearized p*TvLr*DNA-luc (see Figure 4B) to transfect parasite cells and compared the results with transfections using the circular plasmid. Following nucleofection, the cells were used to inoculate 2 independent flasks. After 24 h, one of the flasks was assayed for luciferase activity. Cells transfected with linear DNA showed half the luciferase activity of those transfected with circular DNA (Figure 6A). But after several weeks of selection with G418, the cells transfected with circular DNA started to decay and eventually died whereas those cells transfected with linear DNA formed small clusters and 5 to 10 micro-colonies per flask after 4 weeks of selection. Transfection efficiency using linear DNA was then estimated as 1.5–3.0×10^−7^. The parasites reached confluence 4 weeks later and the supernatants could be transferred into fresh flasks for selection in the presence of G418.

**Figure 7:**
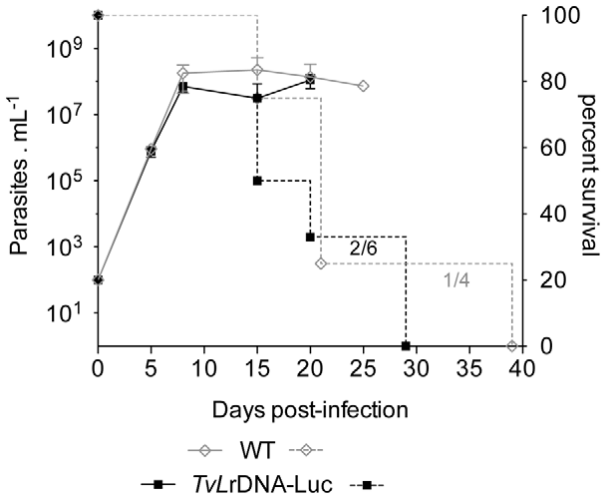
Mutant *Tv*LrDNA-Luc strain displays the same levels of *in vivo* infectivity and virulence as WT *T. vivax*. Four to six Outbred mice were injected intraperitoneally with 10^2^ WT (⋄) or *Tv*LrDNA-Luc (▪) BSF *T. vivax* parasites and parasitemia were followed individually every 5 days. Continous and dotted lines stand respectively for parasitemias and cumulative survival rates, as determined by Kaplan-Meier methodology. Parasitemias are expressed as arithmetic means ± SD. No significance was observed between the curves, as ascertained by the Mantel-Cox log-rank test where *p*>0.5.

After selection, parasite genomic DNA was prepared from two independent cultures stably transfected with p*TvLr*DNA-luc and submitted to PCR using two primer pairs to ascertain whether homologous recombination had occurred, as indicated in the illustration in Figure 6B. Consistently with integration of the p*TvLr*DNA-luc plasmid into the 18 S rDNA region, fragments of the expected size (1.8 kb) were obtained after amplification for both culture DNA (Figure 6C, upper and lower pannels), indicating that homologous recombination had occurred upstream the *TvPRAC* 5′ UTR (Figure 6C, upper pannel) and downstream the *Tv*tubαβ (Figure 6C, lower pannel) regions. The parasites were maintained in axenic TV3 medium for several weeks and luciferase light emission per parasite was shown to be stable over time for at least 24 weeks (Figure 6D) and correlated linearly with the number of parasites on a wide range (>than 4 logs, Figure 6E). Since wild type (WT) metacyclic-like parasites produced in axenic cultures can successfully infect mice, we examined whether this was also the case for the stable metacyclic-like forms developed *in vitro* from the *TvLr*DNA-luc parasite line. Initially, the equivalent to 2×10^6^ *TvLr*DNA-luc metacyclic-like forms obtained from 14-day cultures (see Figure 3C) were used to infect BALB/c mice. At onset of BALB/c parasitemia, a corresponding number of BSF (10^2^) from *TvLr*DNA-luc or WT-infected mice were injected into groups of 4–6 Outbred mice and the parasitemias obtained were compared every 5 days, as previously described [8]. Figure 7 presents the number of BSF recorded during the infection, as well as relative survival in the two groups of mice. It can be seen that parasitemias and survival rates were similar and not significantly different in the mice infected with *TvLr*DNA-luc and those infected with WT parasites. Therefore, after only one passage in mice, the parasitemia kinetics obtained with cultured parasites were similar to those observed with BSF maintained exclusively by serial weekly passages in mice. Similar results were obtained after 2 months and after 12 months of axenic growth, indicating that both WT and mutant *T. vivax* maintained their infectivity after subculturing *in vitro*.

**Figure 8:**
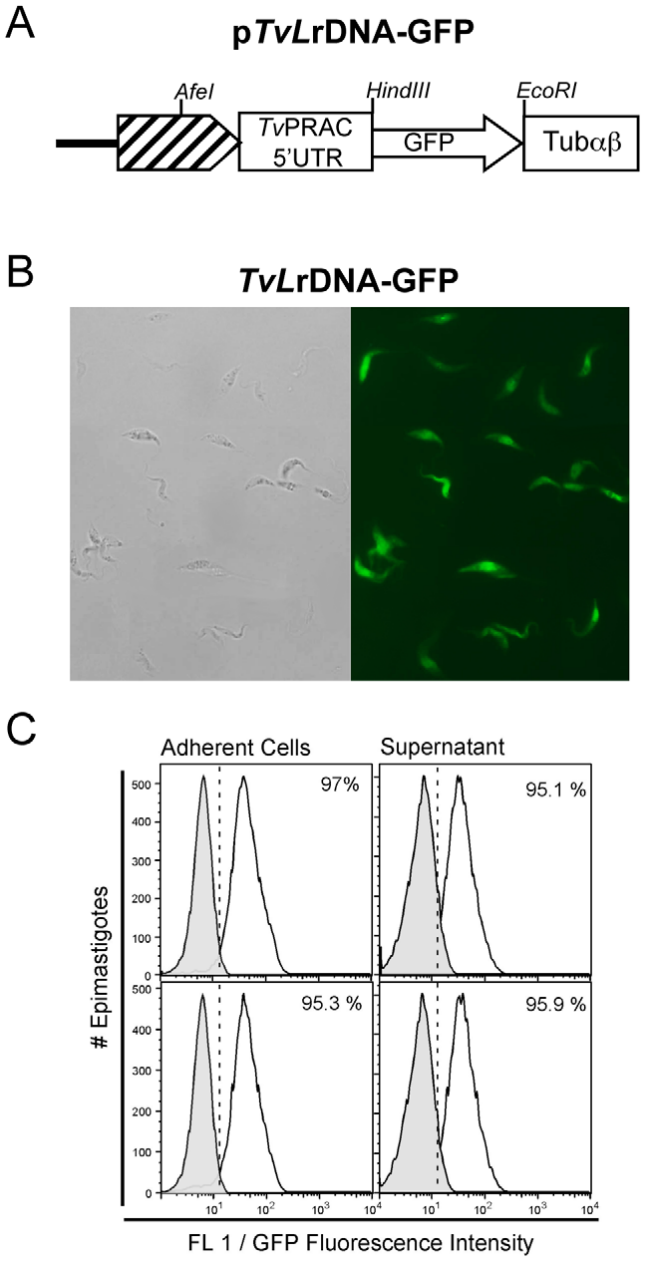
Validation of stably transformed *pTvLr*DNA-GFP parasites. Schematic representation of *pTvLr*DNA-GFP vector construct (A); *TvLr*DNA-GFP parasites from axenic culture supernatants were examined under phase contrast (left panel) and epifluorescence (right panel) microscopy (B). Parasite populations obtained from two independent transfections were cultured and analyzed by flow cytometry. Dead cells were excluded from the acquisition by gating out propidium iodide-stained cells. Parasites were further gated on forward-light scatter/side-light scatter combined gate and the histograms represent the frequency of GFP-expressing *TvLr*DNA-GFP cells (%) as compared to WT, non transformed, control parasites (gray curves) (C).

Ongoing experiments have been performed to further validate the transfection procedures described here above by introducing into the *T. vivax* specific vector another reporter gene. For this aim, *pTvLrDNA-*GFP was constructed by replacing the luciferase gene among the *Tv*PRAC 5′UTR and *Tv*Tubαβ vector sequences by a Green Fluorescent Protein (GFP) cassette excized from the pXS2-GFP ([24], see Methods and Figure 8A). *T. vivax* epimastigotes were transfected with *pTvLrDNA-*GFP using the Amaxa program “×006” and further cultured in TV3 medium at 27°C. Forty eight hours after transfection, G418 (0.5 µg/ml) was added to the cultures to select stable transfectants. Similarly to the parasites transfected with *pTvLrDNA-luc*, massive mortality was observed up to 10 days with the simultaneous growth of stably (*neo^r^*) transformed *T. vivax* that reached confluence four weeks later. The integration of the contruction was validated by PCR using upFrProm/upRrProm and downFrProm/downRrProm couple of primers, as decribed for the luciferase vector (not shown). Figure 8B shows the microscopic observation of recombinant GFP - expressing parasites (*Tv*GFP) obtained from 14 days axenic subcultures. Adherent and supernatant cell populations from two independent cultures were washed in PBS −0.5% glucose and analyzed by cytofluorometry. The FACS analysis shows that both adherent and supernatant *Tv*GFP populations express highly homogenously the GFP (app. 95%), as compared to the absence of fluorescence of WT parasites (Figure 8C). Additional experiments are in progress to evaluate the behavior of *Tv*GFP *in vivo*.

## Discussion

While human African trypanosomosis has drawn the attention of many research groups over the last few decades, less consideration has been given to AAT despite its considerable impact on livestock development and fertility and the economic hardship it causes in several countries. Major breakthroughs have recently been made in the study of *T. congolense*, namely the development and standardization of axenic cultures and the development of transfection techniques [35], [36]. Researchers had showed growing interest in *T. vivax* adaptation to experimental animals from more than twenty years after the encouraging studies of Leeflang in the 1970s, and the description being made of parasite isolates that were infective to rodents [22], [37], [38]. Much attention had also been paid for several years to the development of short-term axenic cultures [14]–[16], [39]. However, few studies conducted over the next two decades quoted these reports on *T. vivax in vitro* growth or parasites obtained in culture, possibly due to methodology inconsistencies and/or difficulties reproducing the complexity of parasite interactions with its host environment [19]. But *T. vivax* still remains a threat for livestock in Africa and South America where the disease is considered as emergent and is consequently the subject of numerous outbreak reports [3], [40].

To gain better insights into the biology of *T. vivax* and its interactions with its mammalian hosts, we recently undertook a detailed study of a pathogenic strain of the parasite that had been isolated in West Africa and stored frozen for several decades. We used this IL 1392 strain to establish novel mouse models of experimental infection and immunopathology [7], [8]. Today, we report herein on how we managed to overcome the lack of genetic tools for analyzing *T. vivax* gene expression and function by standardizing the axenic conditions for epimastigote cultivation of the IL 1392 strain and its *in vitro* differentiation into infectious forms. We also constructed specific vectors appropriate for parasite transgenesis, developed suitable conditions for *T. vivax* transfection and for further selection of transfectants. Moreover, transfection procedures were further validated by the engineering of green fluorescent parasites that stably express the GFP reporter gene. Finally, we carried out an *in vitro* and *in vivo* adaptation of a transgenic *TvLrDNA-luc* parasite line that constitutively expresses the luciferase reporter gene. Our data show that the *TvLrDNA-luc* mutant went through all the *T. vivax* developmental stages *in vitro*, in the same manner as WT parasites, and that metacyclic-like forms of the mutant are infective to immunocompetent mice.

In order to overcome the difficulties found to reproduce culture conditions described in the past we worked to optimize axenic protocols to develop standard conditions of *T. vivax* maintenance and growth. For instance, although fetal calf (FCS) or goat (GS) sera had previously been presented as a essential medium components for trypanosome sustenance and growth *in vitro* [14], [16], our experiments showed that *T.* vivax was unable to grow in culture media supplemented only with FCS or GS. Successful parasite axenic cultures were only possible when a mixture of FCS and goat serum (GS) was used to complement the media, and this resulted in significant BSF attachment, in significant differentiation into epimastigotes and in further parasite growth. Moreover, in the presence of GS, any batch of FCS could be used without affecting parasite differentiation and development. We nonetheless noted that the number of parasites loaded into the cultures had an impact on axenic epimastigote cultivation. For instance, at low densities (<10^6^ cells/ml), *T. vivax* was unable to pursue its developement and the epimastigotes died in a few days without undergoing differentiation or further divisions. Interestingly, and in agreement with previous observations in *T. brucei* [28]–[30], this restriction can be overcome by ensuring that up to one third of the conditioned medium consists of supernatants from former parasite cultures, and in this manner fewer epimastigotes can initially be loaded (i.e. 10^4^ parasites/ml). This observation suggests that conditioned medium contains signaling compounds or growth factors that are released by *T. vivax* in culture and these stimulate and support the proliferation and development of new cells or at least assist in their maintenance under axenic conditions.

It is noteworthy that the axenic culture conditions described herein are suitable for epimastigote differentiation and for the continuous production of metacyclic-like forms. *T. vivax* from the adherent layer differentiate into infective forms without requiring any special medium adaptation, thus mimicking the gradual process of metacyclogenesis in culture. Interestingly, and in contrast to *T. congolense* [35], *T. vivax* parasites that underwent metacyclogenesis *in vitro* from epimastigotes and were then conserved by regular axenic passages for more than one year, retained their infectiveness in immunocompetent mice. And it is of note that the factors involved in metacyclogenesis *per se* are not yet known in nature or described in the literature, for *T. vivax* or *T. congolense*. We cannot foresee whether the optimization of the axenic parasite cultures developed here can be automatically extrapolated to different wild type or other cloned *T. vivax* strains. But, a recent report using *T. congolense* showed that the time period to achieve the adaptation in axenic culture of different parasite strains and derivatives is strain dependant [35]. Nevertheless, previous molecular analysis of IL 1392 has proven the common origin of this and the South American and Asian isolates that phylogenetically pertain to the same clade [41]. It is possible that similarly to IL 1392, strains from the same clade can be cultured using the protocols described herein.

The development in the 1990s of *Trypanosomatid* transfection using Gene Pulser systems [42]–[45] was then adapted to other species [36], [46], [47]. Despite a number of similar transfection parameters shared by all *Kinetoplastidae*, recombination efficiencies and susceptibility to drug selection diverged. Thus, crucial adjustments were necessary to ensure appropriate transfection rates, such as the use of specific parasite regulatory sequences. The vectors we constructed to establish *T. vivax* transfection are based on classical models where foreign genes are placed under the control of species-specific 5′ and 3′ UTRs containing the regulatory sequences required for appropriate gene expression. Consequently, the ribosomal promoter-containing sequence was localized and inserted upstream of the selected transgenes to promote their expression and genomic recombination and thus obtain stably modified transfectants. For purposes of validating our *T. vivax*-specific overexpressing vector, we compared the archetypal Gene Pulser transfection system with Amaxa nucleofection technology. Amaxa technology has been reported, with *T. brucei*, to greatly increase the number of transfectants obtained [34], [48]. In line with this, an initial series of experiments showed that Amaxa protocols had two major advantages over Gene Pulser conditions: they improved transient transfection efficiency and increased parasite viability and adhesion to the surface of the culture flask. Subsequent experiments showed that transfection of the *T. vivax*-derived circular vector was unable to generate stable transfectants. This may suggest that *T. vivax* is not able to maintain episomal DNA, unlike *T. brucei* and *T. congolense* that carry out particular sequences (i.e. *parp*) that promote episomal mantainance [36], [48]. Alternatively, *T. vivax* circular DNA may be integrated but less efficiently than linear plasmids, resulting in a smaller number of live parasites that do not survive culture conditions. By contrast, when the linearized vector was used for the transfections, this generated stable transfectants that showed appropriate integration of the foreign gene into the ribosomal promoter region. This result shows that the linearized vector facilitates integration, as already shown for other kinetoplastids [33], [49].

In conclusion, the *T. vivax* strain generated in our studies and stably expressing a luciferase reporter gene will be very useful for characterizing the *in vivo* infectious process and for validating the effectiveness of drug candidates in medium or high-throughput screening tests. Bioluminescent *T. vivax* will also certainly prove useful in enhancing our knowledge of the different aspects of parasite development, the acquisition of virulence and the triggering of pathology. However, cellular trafficking and localization of a given stage specific parasite protein may be promptly assessed by parasite engineered with fluorescent specific vector described here, where the current GFP cassette would be replaced by a gene of interest fused to the GFP reporter. Under the same promoter elements, transgenic parasites should express the fusion protein linked to GFP and easily identified. The work described herein has therefore developed the first specific genetic tools for the study of *T. vivax* biology and opens up new possibilities for the study of experimental *Nagana*, particularly the expression and regulation of critical genes implicated in the parasite's evasion of the host immune system. Additionaly, our work also paves the way for the development of more sophisticated tools to reduce the expression of parasite genes by inducible RNAi or by conventional gene knockout based on homologous recombination.
